# Impact of Partial Penectomy and Glansectomy on Couples’ Sexual Function

**DOI:** 10.1590/S1677-5538.IBJU.2025.0314

**Published:** 2025-07-30

**Authors:** Angelo Maurílio Fosse, Mauro Romero Leal Passos, Alex Schul, Daniel Carvalho Ribeiro, José Genilson Alves Ribeiro, Ronielly Bozzi, Rodrigo Barros

**Affiliations:** 1 Universidade Federal Fluminense Serviço de Urologia do Hospital Universitário Antônio Pedro Niterói RJ Brasil Serviço de Urologia do Hospital Universitário Antônio Pedro, Universidade Federal Fluminense - UFF, Niterói, RJ, Brasil

**Keywords:** Penile Neoplasms, Sexual Health, Sexual Behavior

## Abstract

**Objective:**

To evaluate the sexual function of couples after surgeries to treat penile cancer.

**Material and Methods:**

Patients who underwent partial penectomy or glansectomy at a cancer center, between April 2014 and January 2024, and their partners were interviewed only once, at least six months after surgery, through individually conducted interviews. We used a semi-structured questionnaire, the Six-Item Female Sexual Function Index Scoring (FSFI-6) questionnaire to assess feminine sexual function and the Erection Hardness Score (EHS) and The International Index of Erectile Function-5 (IIEF-5) questionnaire to evaluate erectile function.

**Results:**

Fourteen couples were included in this study. Five patients underwent glansectomy and nine underwent partial penectomy. All of male patients remained sexually active post-procedure. Of the ten who completed the IIEF-5, five (50%) experienced mild erectile dysfunction. Among partners, twelve (85.7%) continued sexual activity. Of the ten partners who completed the FSFI-6, five (50%) had sexual dysfunction. The size of the penile stump was not correlated with satisfaction of the couples. Four (28.6%) patients adopted adaptive strategies, as engaging in sexual activity in a dark environment and use of silicone penile substitutes. All participants expressed dissatisfaction with the medical information provided by their doctors regarding sexuality after penile surgery.

**Conclusions:**

Many couples described sexual dysfunctions after PC surgeries. However, the majority of couples reported maintaining an active sexual life and the majority of the partners reported achieving orgasm. Unfortunately, counseling by healthcare professionals of these couples about sexual health after surgery to treat PC is inadequate.

## INTRODUCTION

Penile cancer (PC) is rare in industrialized countries, with a higher incidence in regions of Africa, Asia, and South America (1). In Brazil, the incidence varies from 2.9 to 6.8 per 100,000, affecting 5.7% of male neoplasms in populations residing in the Northeast region (2, 3). Squamous cell carcinoma is the most common histological type, accounting for over 95% of malignant penile neoplasms (4).

Early-stage PC (T1–T2) can be conservatively treated with 5-FU, laser, circumcision, or organ-sparing surgery like glans resurfacing and local excision with grafting recommended for glans tumors <4 cm without deep invasion. Invasive PC requires tumor excision with clear margins. T2 glans tumors are treated with glansectomy and grafts. Larger or deeper lesions may need partial penectomy, while total penectomy with perineal urethrostomy is reserved for extensive or advanced cases (5). Prophylactic lymphadenectomy is a viable procedure for selected patients at high risk of metastasis. However, no definitive predictive biomarker of inguinal lymph node metastasis has yet been established and there are many challenges to achieve this goal (6)

Penile surgeries offer good cancer control but impact body image, self-esteem, sensitivity, and sexual function (7, 8). Partial penectomy often leads to erectile dysfunction (ED) and shame over penile size and glans loss, hindering return to sexual activity (9). However, literature is scarce regarding the impact on the sexual life of couples after penectomy or organ sparing surgery, especially concerning the quality of sexual life for partners.

We believe that majority of couples maintain satisfactory sexual function after penectomy or glansectomy and counseling by healthcare professionals of these couples about sexual health is inadequate.

The objective of this study was to assess the impact of partial penectomy or glansectomy on sexual function in patients and partners.

## MATERIALS AND METHODS

This study was approved by the Research Ethics Committee of our university (IRB 66905623.0.0000.5243). In this cross-sectional study, patients who underwent partial penectomy or glansectomy at a cancer center, between April 2014 and January 2024, and their partners were invited to participate. They were interviewed only once, at least six months after surgery, through individually conducted interviews. Each participant responded to a semi-structured questionnaire. Demographic and clinical data were retrospectively collected from interviews and medical records. Men were queried about erectile function using validated questionnaires Erection Hardness Score (EHS) (9), regarding penile rigidity and The International Index of Erectile Function-5 (IIEF-5) (10) concerning the post-operative period. No patients reported the continuous use of phosphodiesterase-5 inhibitors. Additionally, the length of the flaccid penile stump from the pubic symphysis to the distal end of the penis was measured using a ruler by a single observer (11). The partners of these patients had their sexual function evaluated using the Six-Item Female Sexual Function Index Scoring (FSFI-6) questionnaire (12). Exclusion criteria included partners with painful urogenital disease, ongoing incurable cancer, prior extensive pelvic treatment, male partners, couples with less than one year of relationship at the time of surgery, and cases of total penectomy or glans resurfacing.

Data were analyzed using means, standard deviations, frequencies, and percentages. Spearman's correlation was applied, with p < 0.05 considered significant. Analyses were performed in MedCalc software® (MedCalc software Ltd, Belgium), version 20.006.

## RESULTS

This study included 14 couples in which the man had undergone partial penectomy (n = 9) or glansectomy (n = 5) for PC surgical treatment. The duration of the relationships ranged from 3 to 50 years, with an average of 22.3±12.9 years. All the couples were married and reported being heterosexual. The average age of the men was 58.8±13.4 years while the average age of the female partners was 56.4±11.6 years. Table-1 details sociodemographic and clinical characteristics of couples and their sexual function scores.

**Table 1:** Sociodemographic and Clinical Characteristics of Couples Affected by Penile Cancer and Their Sexual Function Scores.

Couple	Duration of relationship (y)	Patient			Partner			Post-operative time of interview (months)	Surgery type	Pre-operative EHS	Post-operative EHS	IIEF-5	FSFI-6	Penile length (cm)
		Age	Education	Religion	Age	Education	Religion							
**1**	15	53	HS	E	48	PS	E	22	PP	4	2	20	25	6.5
**2**	50	74	PS	E	52	PS	E	90	PP	4	3	19	22	5
**3**	22	45	PS	E	46	PS	E	23	G	4	4	24	25	7
**4**	3	62	PS	C	59	HS	C	45	G	4	4	24	24	8.5
**5**	40	66	PS	C	67	PS	C	41	G	4	3	NA	NA	4
**6**	40	86	HS	C	87	HS	C	29	PP	2	2	10	24	5
**7**	20	52	PS	E	53	PS	E	24	PP	4	1	9	15	6
**8**	21	48	PS	C	48	PS	C	75	PP	3	2	NA	NA	4
**9**	20	50	PS	C	55	HS	E	25	PP	4	4	NA	NA	3.5
**10**	20	60	PS	C	56	PS	C	25	PP	4	4	NA	NA	7
**11**	15	41	HS	A	43	CO	C	78	G	3	3	23	9	9
**12**	10	47	HS	E	62	PS	C	30	PP	3	3	17	8	8.5
**13**	12	61	PS	A	47	PS	S	18	G	4	4	20	19	6.5
**14**	24	41	PS	E	43	PS	E	15	PP	3	3	18	18	4
General mean±SD								38.5±24.5		3.6±0.6	3.0±1.0	18.4±5.3	18.9±6.4	6.0±1.8
**Glansectomy (G) mean±SD**										3.8±0.4	3.6±0.5	22.75±1.9	19.25±7,3	7.0±2.0
**Partial penectomy (PP) mean±SD**										3.4±0.7	2.7±1.0	15.5±4.8	18.7±6.4	5.5±1.6

SD = standard deviation; HS = high school; PS = primary school; CO = college; E = evangelical; C = catholic; A = atheist; S = spiritualist; NA = not applicable; G = glansectomy; PP = partial penectomy

Of the cases, 13 (92.9%) were localized PC and 1 (7.1%) had advanced PC. Localized PC refers to tumors that are confined to the penis without evidence of lymphatic or distant metastasis, typically classified as T1 and T2 lesions in the TNM staging system. Advanced PC includes tumors that have extended beyond the primary site, involving deeper structures such as the corpora cavernosa or urethra (T3, T4), regional lymph nodes (N1-N3), or distant metastasis (M1) (13). The surgical and anatomopathological findings are better detailed in Table-2.

**Table 2:** Description of Surgical and Pathological Results in Penile Cancer and Postoperative Sexual Behavior of the Couple.

Surgery type		N (%)
**Glansectomy**		5 (35.7%)
**Partial penectomy**		9 (64.3%)
**Histological Subtype**		
	Squamous	13 (92.9%)
	Verrucous	1 (7.1%)
**Histological Grade**		
	I (well-differentiated)	4 (28.6%)
	II (moderately differentiated)	9 (64.3%)
	III (undifferentiated)	1 (7.1%)
**TNM Classification**		
	**Primary Tumour**	
	T1	5 (35.7%)
	T2	8 (57.2%)
	T3	1 (7.1%)
	**Regional Lymph Nodes**	
	N0	12 (85.7%)
	N1	2 (14.3%)
	**Distant Metastasis**	
	M0	14 (100%)
	M1	0
	**Lymphadenectomy**	
	Yes	7 (50%)
	No	7 (50%)
**Sexual intercourse**		Patient/Partner
	Yes	14 (100%)/12 (85.7%)
	No	0/2 (14.3%)
**Preferred type of sexual intercourse**		
	Vaginal	5 (35.7%)/3 (25%)
	Digital stimulation	1 (7.1%)/1 (8.3%)
	Vaginal/Anal	1 (7.1%)/1 (8.3%)
	Vaginal/Oral/Digital Stimulation	3 (21.5%)/2 (16.7%)
	Oral / Digital Stimulation	3 (21.5%)/1 (8.3%)
	Oral	0/2 (16.7%)
	Vaginal/Digital Stimulation	1 (7.1%)/2 (16.7%)
**Preferred vaginal sex position**		
	Man on top	6 (60%)/5 (62.5%)
	Woman on top	2 (20%)/1 (12.5%)
	Doggy-style position	1 (10%)/1 (12.5%)
	Side position	1 (10%)/1 (12.5%)
**Orgasm**		
	Yes	13 (92.9%)/8 (66.7%)
	No	1 (7.1%)/4 (33.3%)
**Extra-marital relationship**		
	Yes	1 (7.1%)/0
	No	13 (92.9%)/14 (100%)

Nine patients underwent partial penectomy (64.3%) and five underwent glansectomy (35.7%) with split-thickness skin grafts utilized for neoglans reconstruction. Among the 6 patients classified as primary tumor T1, 4 underwent partial penectomy and 2 underwent glansectomy.

Regarding penile size in the flaccid state, the length of the penile stump among these patients ranged from 3.5 to 9 cm (mean±SD: 6.0±1.8cm). Only one patient of the cohort (7.1%) presented with a stump <4 cm, while the patient with the largest penile stump in the series had undergone glansectomy. All the men reported maintaining sexual activity after the surgery. Of the 14 patients in this study, four of them (28.6%) do not engage in penetrative sexual intercourse, opting instead for digital and oral stimulation. Among these four patients, three (75%) underwent partial penectomy and one (25%) underwent glansectomy. Nine patients of this study (64.3%) exhibited a degree of good satisfaction according to the semi-structured questionnaire data, with only one (7.1%) man who underwent partial penectomy reporting an inability to achieve orgasm. The mean reported sexual frequency was 4.8±4.1 encounters per month. One (7.1%) patient disclosed engaging in extramarital relations post-glansectomy. Of the 14 patients, 10 completed the IIEF-5 questionnaire, as it is applicable exclusively to those engaging in penetrative sexual activity. Of these, five (50%) developed mild ED (IIEF-5 between 17 and 21 points), two (20%) developed moderate ED (IIEF-5 between 8 and 11 points), and three others (30%) did not exhibit ED (IIEF-5 ≥ 22). When analyzing the surgery subgroup, glansectomy did not exhibit ED (mean IIEF-5±SD 22.75±1.9) while partial penectomy group developed mild ED (mean IIEF-5±SD 15.5±4.8). Eleven (78.6%) out of 14 patients undergoing reported satisfactory penile rigidity according EHS (score 3 or 4), independently of the specific surgical technique employed. However, EHS score was higher in the glansectomy group (3.6±0,5) than in the partial penectomy group (2.7±1) (Table-1).

In regard to the sexual function of their partners, twelve women (85.7%) reported continuing to engage in sexual activity, with discrepancies noted in the male responses in two cases. The average frequency of intercourse reported by them was 3.2±3.0 times a month. Only four (33.3%) women reported not achieving orgasm, and 6 (50%) exhibited a degree of regular satisfaction according to the semi-structured questionnaire data. Of the 12 women with an active sexual life, 3 (25%) reported good sexual satisfaction, 6 (50%) reported regular satisfaction, and 3 (25%) reported poor sexual satisfaction. Only 2 women reported experiencing pain during sexual intercourse. Despite the pain, both women reported achieving orgasm. Among the 10 women who engaged in vaginal intercourse and were able to respond to the FSFI-6 questionnaire, 5 (50.0%) experienced sexual dysfunction (FSFI-6 ≤ 20), primarily related to low sexual satisfaction and difficulty achieving orgasm. The degree of female sexual dysfunction was similar between the surgical type groups (Table-1).

Vaginal intercourse was the preferred type of sexual activity reported by men (35.7%) and their partners (25%). Regarding preferred sexual positions for vaginal intercourse, of the 10 men engaging in this practice, 6 (60%) reported a preference for the man-on-top position, 2 (20%) for the woman-on-top position, 1 (10%) for the doggy-style position, and 1 (10%) for the side position. Although 10 patients reported engaging in penetrative sexual intercourse, 8 partners reported the same. Among the 8 women reporting vaginal sexual activity, 5 (62.5%) preferred the man-on-top position, 1 (12.5%) preferred the woman-on-top position, 1 (12.5%) preferred the doggy-style position, and 1 (12.5%) preferred the side position. Four men (28.6%) adopted adaptive strategies. Two of them (50%) started having sexual relations in a dark environment, and the other 2 (50%) began using a silicone penile substitute for vaginal penetration. There was no statistically significant correlation between penile stump size and sexual satisfaction (p=0.44) or type of sexual activity (p=0.81). Moreover, there was no significant association between the type of surgical technique (e.g., glansectomy vs. partial penectomy) and either sexual satisfaction or penile stump length. Data on the couple's sexual behavior after the surgeries are described in Table-2.

In this study, all patients and their partners reported not having been adequately informed about post-surgical sexuality, as answered in the semi-structured questionnaire.

## DISCUSSION

PC treatment may be conservative or surgical. Organ-sparing methods preserve function and appearance, with surgical choice based on stage, margins, and patient preference (5, 13, 14). In this study, partial penectomy was the most frequently performed surgical procedure (64.3%), followed by glansectomy (35.7%).

Surgery and cancer affect body image, confidence, sensitivity, and sexual activity, often causing performance anxiety, especially after penectomy, with rates up to 90% (7–9, 15, 16). Despite this impact, few studies assess male sexuality, and only one includes both partners (17).

PC is a disease that usually affects patients aged 50 and over (18–20). In this study, the mean age of the patients was 58 years and of the partners was 56 years. Considering the 14 men who have an active sexual life, the level of sexual satisfaction was deemed good for 9 (64.3%) of them, regular for 4 (28.6%), and poor for only one (7.1%). All of the men reported maintaining sexual activity after surgery, with an average reported sexual frequency of 4.8 encounters per month. According to a literature review involving 128 penectomized patients using 13 different quality of life assessment instruments, an incidence of 36% of cases without sexual activity was observed (8).

Partial penectomy often leads to fewer orgasms and greater concern with penile appearance, especially due to glans absence, even without ED or partner issues (9, 21, 22). In our study, only one man, unable to perform penetration, reported not having orgasms. Within our series, no differences in sexual satisfaction were observed based on the specific surgical technique employed. Nonetheless, Falcone et al. reported that glans resurfacing seems to provide better sexual outcomes when compared to other organ sparing approaches (14). ED is common after partial penectomy. Monteiro et al. found a 62% incidence, linked to shorter shaft, positive nodes, and age (16). Psychological factors like shame over penile size and glans loss also hinder sexual activity (17).

Penetration influences female sexual satisfaction, with pain or discomfort reducing it (23). Strong pelvic floor muscles are linked to better sexual function (24). In our study, most partners showed a preference for sexual intercourse with penetration, with a preference for the man-on-top position. However, we know that penetrative sex is not the only aspect of a satisfactory sexual relationship (17). Nevertheless, measuring the degree of sexual satisfaction in women is challenging due to biological, psychological, physiological, and cultural variables that can influence the final satisfaction level (25). Therefore, we applied the validated FSFI-6 questionnaire with the aim of using a simpler and reliable assessment tool. In our sample, five women had scores indicative of sexual dysfunction. The partner with the highest level of sexual dysfunction has a partner with of the longest penile stump, measuring 8.5 cm. These findings in this small cohort support the literature that larger penis size and penetration are not unique of female sexual satisfaction (17). Interestingly, the partners who achieved the highest scores on the FSFI-6 questionnaire were those whose partners underwent partial penectomy and glansectomy, with penile stumps measuring 6.5 cm and 7 cm, respectively.

According to a study that applied the FSFI-6 questionnaire to 737 women aged 40 to 55, a prevalence of 53.5% of female sexual dysfunction was observed. Older age, menopausal symptoms, and age at menopause onset were inversely correlated with sexual function (12). Additionally, four women reported not having orgasms, all with a regular satisfaction level. Despite not achieving orgasm, the average sexual frequency was 3.2 encounters per month.

Adaptive measures are often utilized by these couples. A very common condition, which was also observed in our study, is engaging in sexual activity in a dark environment, indicating protection from visual exposure to the mutilated genitals. Adopting a more favorable position for penetration proved to be a simple and effective action for one of the couples. However, in general, the use of penile stretching sleeves or external penile prostheses was a common tactic employed, including in patients after total penectomy (7, 26). On the other hand, some couples reported engaging in non-penetrative sexual relations, using alternative stimulation techniques, discovering new erogenous zones, and developing new forms of intimacy and emotional connection. Previous studies have shown that, with the maintenance of the partnership, there is a high possibility that post-penectomy sexual relations will be of similar quality to pre-penectomy relations (17, 27, 28). Bhat et al. evaluated the sexual lives of 12 couples after penectomy, including two cases of total penectomy. The authors observed that one patient experienced orgasm through stimulation of the penectomy scar at the base of the penis. Additionally, patients and their partners reported accepting masturbation as a means of achieving sexual satisfaction (17).

PC is a rare tumor, and few urologists have personal experience with the postoperative sexual life of these patients. The lack of data in the medical literature in this area leads physicians to underestimate the sexual performance of patients, who often retain the same ability for intercourse as before surgery (29). In this study, all patients and their partners reported not being adequately informed about post-surgical sexuality. Adequate preoperative counseling by the treating physician can assist in post-treatment adjustment regarding sexuality, for both patients and their partner (17). Additionally, medical support before surgery and the continuity of professional support can improve sexual satisfaction rates for couples who have undergone penectomy (30, 31).

This study has some limitations. Self-reported data may include omissions, and lack of preoperative IIEF-5 limits ED assessment. The small sample size is our main limitation. However, to our knowledge, this is the second study published in the literature evaluating the impact on couple's sexual function after penectomy with a larger sample and deeper partner analysis. Moreover, the purpose of this study was achieved, and we identified that the majority of couples maintain an active sexual life with satisfactory sexual function, highlighting an important information for the field of Onco-sexology.

## CONCLUSION

Many couples described sexual dysfunctions after PC surgeries. Mild to moderate ED was common. However, the majority of couples reported maintaining an active sexual life and the majority of the partners reported achieving orgasm and the ability to maintain satisfactory sexual function. The size of the penile stump was not correlated with satisfaction of the couples. Adaptive behaviors were used with the goal of ameliorating the impact of the masculine mutilation and improving the sexual satisfaction.

Counseling by healthcare professionals of these couples about sexual health after surgery to treat PC is inadequate. Therefore, it is important to develop programs with a multi-professional approach about sexual health to meet unsatisfied needs of couples. Finally, multicentric studies and larger sample sizes are needed to improve the understanding of the impact of penectomy on couples’ sexual function.

## References

[1] Siegel RL, Miller KD, Jemal A (2016). Cancer statistics, 2016. CA Cancer J Clin.

[2] Christodoulidou M, Sahdev V, Houssein S, Muneer A (2015). Epidemiology of penile cancer. Curr Probl Cancer.

[3] Favorito LA, Nardi AC, Ronalsa M, Zequi SC, Sampaio FJ, Glina S (2008). Epidemiologic study on penile cancer in Brazil. Int Braz J Urol.

[4] Tang V, Clarke L, Gall Z, Shanks JH, Nonaka D, Parr NJ (2014). Should centralized histopathological review in penile cancer be the global standard?. BJU Int.

[5] Muneer A, Bandini M, Compérat E, De Meerleer G, Fizazi K, ESMO Guidelines Committee (2024). Penile cancer: ESMO–EURACAN Clinical Practice Guideline for diagnosis, treatment and follow-up. ESMO Open.

[6] Campos MAG, Teixeira AALJ, Calixto JRR, Larges JS, Pinho JD, Silva GEB (2023). Predictive histopathological factors of nodal metastasis in penile cancer. Int Braz J Urol.

[7] Anderson D, Razzak AN, McDonald M, Cao D, Hasoon J, Viswanath O (2022). Mental Health in Urologic Oncology. Health Psychol Res.

[8] Maddineni SB, Lau MM, Sangar VK (2009). Identifying the needs of penile cancer sufferers: a systematic review of the quality of life, psychosexual and psychosocial literature in penile cancer. BMC Urol.

[9] Romero FR, Romero KR, Mattos MA, Garcia CR, Fernandes Rde C, Perez MD (2005). Sexual function after partial penectomy for penile cancer. Urology.

[10] Santos Pechorro P, Martins Calvinho A, Monteiro Pereira N, Xavier Vieira R (2011). Validação de uma versão portuguesa do Índice Internacional de Função Eréctil-5 (IIEF-5). Rev Int Androl.

[11] Falcone M, Bettocchi C, Carvalho J, Ricou M, Boeri L, Capogrosso P (2024). European Association of Urology Guidelines on Penile Size Abnormalities and Dysmorphophobia: summary of the 2023 guidelines. Eur Urol Focus.

[12] Dall’Agno ML, Ferreira CF, Ferreira FV, Pérez-López FR, Wender MCO (2019). Validation of the six-item Female Sexual Function Index in middle-aged Brazilian women. Rev Bras Ginecol Obstet.

[13] Bologna E, Licari LC, Franco A, Ditonno F, Manfredi C, De Nunzio C (2024). Characteristics, trends, and management of penile cancer in the United States: a population-based study. Urol Oncol.

[14] Falcone M, Preto M, Gül M, Şahin A, Scavone M, Cirigliano L (2024). Functional outcomes of organ sparing surgery for penile cancer confined to glans and premalignant lesions. Int J Impot Res.

[15] Barros R, Favorito LA, Nahar B, Almeida R, Ramasamy R (2023). Changes in male sexuality after urologic cancer: a narrative review. Int Braz J Urol.

[16] Monteiro LL, Skowronski R, Brimo F, Carvalho PDC, Vasconcelos RAL, Pacheco C (2021). Erectile function after partial penectomy for penile cancer. Int Braz J Urol.

[17] Bhat GS, Nelivigi G, Barude V, Shastry A (2018). Sexuality in surgically treated carcinoma penis patients and their partners. Indian J Surg.

[18] Coelho RWP, Pinho JD, Moreno JS, Garbis D, do Nascimento AMT, Larges JS (2018). Penile cancer in Maranhão, Northeast Brazil: the highest incidence globally?. BMC Urol.

[19] Koifman L, Vides AJ, Koifman N, Carvalho JP, Ornellas AA (2011). Epidemiological aspects of penile cancer in Rio de Janeiro: evaluation of 230 cases. Int Braz J Urol.

[20] Korkes F, Rodrigues AFS, Baccaglini W, Timóteo F, Slongo J, Spiess P (2020). Penile cancer trends and economic burden in the Brazilian public health system. Einstein (Sao Paulo).

[21] Kieffer JM, Djajadiningrat RS, van Muilekom EA, Graafland NM, Horenblas S, Aaronson NK (2014). Quality of life for patients treated for penile cancer. J Urol.

[22] Sosnowski R, Kulpa M, Kosowicz M, Wolski JK, Kuczkiewicz O, Moskal K (2016). Quality of life in penile carcinoma patients – post-total penectomy. Cent European J Urol.

[23] Rosen R, Brown C, Heiman J, Leiblum S, Meston C, Shabsigh R (2000). The Female Sexual Function Index (FSFI): a multidimensional self-report instrument for the assessment of female sexual function. J Sex Marital Ther.

[24] Magno LDP, Fontes-Pereira AJ, Nunes EFC (2011). Avaliação quantitativa da função sexual feminina correlacionada com a contração dos músculos do assoalho pélvico. Rev Pan-Amaz Saúde.

[25] Hentschel H, Alberton DL, Capp E, Goldim JR, Passos EP (2007). Validação do Female Sexual Function Index (FSFI) para uso em língua portuguesa. Rev HCPA.

[26] van Dongen J, de Heus E, Eickholt L, Schrieks M, Zantingh I, Brouwer OR (2022). Challenges and controversies patients and (health care) professionals experience in managing vaginal, vulvar, penile or anal cancer: The SILENCE study. Eur J Cancer Care (Engl).

[27] Sosnowski R, Wolski JK, Kulpa M, Zietalewicz U, Kosowicz M, Kalinowski T (2017). Assessment of quality of life in patients surgically treated for penile cancer: impact of aggressiveness in surgery. Eur J Oncol Nurs.

[28] Sosnowski R, Wolski JK, Zietalewicz U, Szymański M, Bakuła AR, Demkow T (2019). Assessment of selected quality of life domains in patients who have undergone conservative or radical surgical treatment for penile cancer: an observational study. Sex Health.

[29] Opjordsmoen S, Waehre H, Aass N, Fossa SD (1994). Sexuality in patients treated for penile cancer: patients’ experience and doctors’ judgement. Br J Urol.

[30] Barros R, Fosse AM, Schul A, Mangas G, Teixeira CHDS, Ninis T, Carvalho JPM, Favorito LA (2025). Impact of Diagnosis of Urologic Cancer on Male Sexuality and Patients’ Perceptions of Health Professional's Approach. Int Braz J Urol.

[31] Sansalone S, Silvani M, Leonardi R, Vespasiani G, Iacovelli V (2017). Sexual outcomes after partial penectomy for penile cancer: results from a multi-institutional study. Asian J Androl.

